# Exertional Heat Stroke, Modality Cooling Rate, and Survival Outcomes: A Systematic Review

**DOI:** 10.3390/medicina56110589

**Published:** 2020-11-05

**Authors:** Erica M. Filep, Yuki Murata, Brad D. Endres, Gyujin Kim, Rebecca L. Stearns, Douglas J. Casa

**Affiliations:** 1 Korey Stringer Institute, University of Connecticut, Storrs, CT 06269-1110, USA; endres.brad@gmail.com (B.D.E.); gyujin.kim@uconn.edu (G.K.); rebecca.stearns@uconn.edu (R.L.S.); douglas.casa@uconn.edu (D.J.C.); 2 Graduate School of Education and Human Development, Nagoya University, Furo-cho, Chikusa-ku, Nagoya 464-8601, Japan; murata.yuki@c.mbox.nagoya-u.ac.jp

**Keywords:** prehospital emergency care, clinical protocols, body temperature, hyperthermia

## Abstract

*Background and Objectives:* The purpose of this systematic review is to synthesize the influence cooling modality has on survival with and without medical complications from exertional heat stroke (EHS) in sport and military populations. *Methods and Materials:* All peer-reviewed case reports or series involving EHS patients were searched in the following online databases: PubMed, Scopus, SPORTDiscus, Medline, CINAHL, Academic Search Premier, and the Cochrane Library: Central Registry of Clinical Trials. Cooling methods were subdivided into “adequate” (>0.15 °C/min) versus “insufficient” (<0.15 °C/min) based on previously published literature on EHS cooling rates. *Results*: 613 articles were assessed for quality and inclusion in the review. Thirty-two case reports representing 521 EHS patients met the inclusion criteria. Four hundred ninety-eight (498) patients survived EHS (95.58%) and 23 (4.41%) patients succumbed to complications. Fischer’s Exact test on 2 × 2 contingency tables and relative risk ratios were calculated to determine if modality cooling rate was associated with patient outcomes. EHS patients that survived who were cooled with an insufficient cooling rate had a 4.57 times risk of medical complications compared to patients who were treated by adequate cooling methods, regardless of setting (RR = 4.57 (95%CI: 3.42, 6.28)). *Conclusions:* This is the largest EHS dataset yet compiled that analyzes the influence of cooling rate on patient outcomes. Zero patients died (0/521, 0.00%) when treatment included a modality with an adequate cooling rate. Conversely, 23 patients died (23/521, 4.41%) with insufficient cooling. One hundred seventeen patients (117/521, 22.46%) survived with medical complications when treatment involved an insufficient cooling rate, whereas, only four patients had complications (4/521, 0.77%) despite adequate cooling. Cooling rates >0.15 °C/min for EHS patients were significantly associated with surviving EHS without medical complications. In order to provide the best standard of care for EHS patients, an aggressive cooling rate >0.15 °C/min can maximize survival without medical complications after exercise-induced hyperthermia.

## 1. Introduction

Exertional Heat Illness (EHI) is a major concern among athletes, laborers, and warfighters throughout the world. EHIs are most likely occur in hot and humid environments, which are often experienced in sporting events and military training/exercises [1,2,3,4]. However, EHIs can also occur with intense physical activity in the absence of extreme environmental conditions. EHIs include exercise-associated muscle cramps (EAMCs), heat syncope, heat exhaustion, and exertional heat stroke (EHS). EHS is considered a catastrophic injury due to the risk of death if not treated appropriately [2,3,5,6]. In the literature, EHS diagnostic criteria includes central nervous system (CNS) dysfunction in addition to a body temperature above 40.5 °C (105 °F) [2,3,5]. If quickly and appropriately treated, EHS is survivable without medical complications [2,3,5,6,7,8,9,10,11].

Maximizing EHS survival hinges on valid measures of internal body temperature, cooling modality, and prehospital treatment. The military setting provides a unique challenge to on-site cooling due to the nature of the location and work performed by warfighters. In sport, best practices for treating EHS include obtaining a valid body temperature, cold-water immersion (CWI), and cooling on site prior to transport to an emergency department [2,3,5,6,7,9,11,12].

Soldiers and athletes face unique and harsh environmental conditions as they work towards their specific goal. Military duty requires individuals to work in conditions where heat dissipation can be impeded by wearing protective armor, carrying equipment, and the nature of the physical environment [12,13]. Between 2008 and 2018, there were 4188 cases of EHS in the United States Armed Forces [14,15,16,17,18,19,20,21,22,23]. Athletic events, such as American football, also require protective equipment that can decrease a person’s ability to dissipate heat. While American football is not equitable to active military duty, the physical stress of the game and its protective equipment can pose a challenge to athletes when conditioning, practicing, and competing during times of increased heat and relative humidity. Road races, marathons, ultra-marathons, etc., amass thousands of participants, all of whom have varying medical histories and comorbidities that can increase risk for EHS [1,2,6,9,10,24].

Fatalities from EHS have been extensively reported in the literature across a variety of settings when best practices are not utilized [25,26,27,28,29]. In sport, EHS is the third leading cause of death after cardiac conditions and brain injury [2,6]. It is the leading cause of indirect fatality in athletics [2,3,6,30]. The epidemiology of EHS fatalities and the efficacy of CWI in decreasing body temperature has been heavily examined in the military and organized sports settings [9,11,30,31,32,33,34,35]. However, these studies have been performed respectively and the findings have yet to be integrated. Consequently, there remains a lack of understanding of EHS survival rates when rapid recognition and appropriate treatment are implemented within military and sport settings. To our knowledge, no systematic review has examined EHS survival outcomes in sport or military settings and the influence of cooling rate on patient outcomes. The purpose of this systematic review is to synthesize the influence cooling modality has on the prevalence of survival with and without medical complications (MC) from EHS patients from both sport and military populations. We aim to identify the total increase in risk of medical complications from EHS related to treatment used for patients in these populations.

## 2. Materials and Methods

### 2.1. Search Strategy

**Figure 1:**
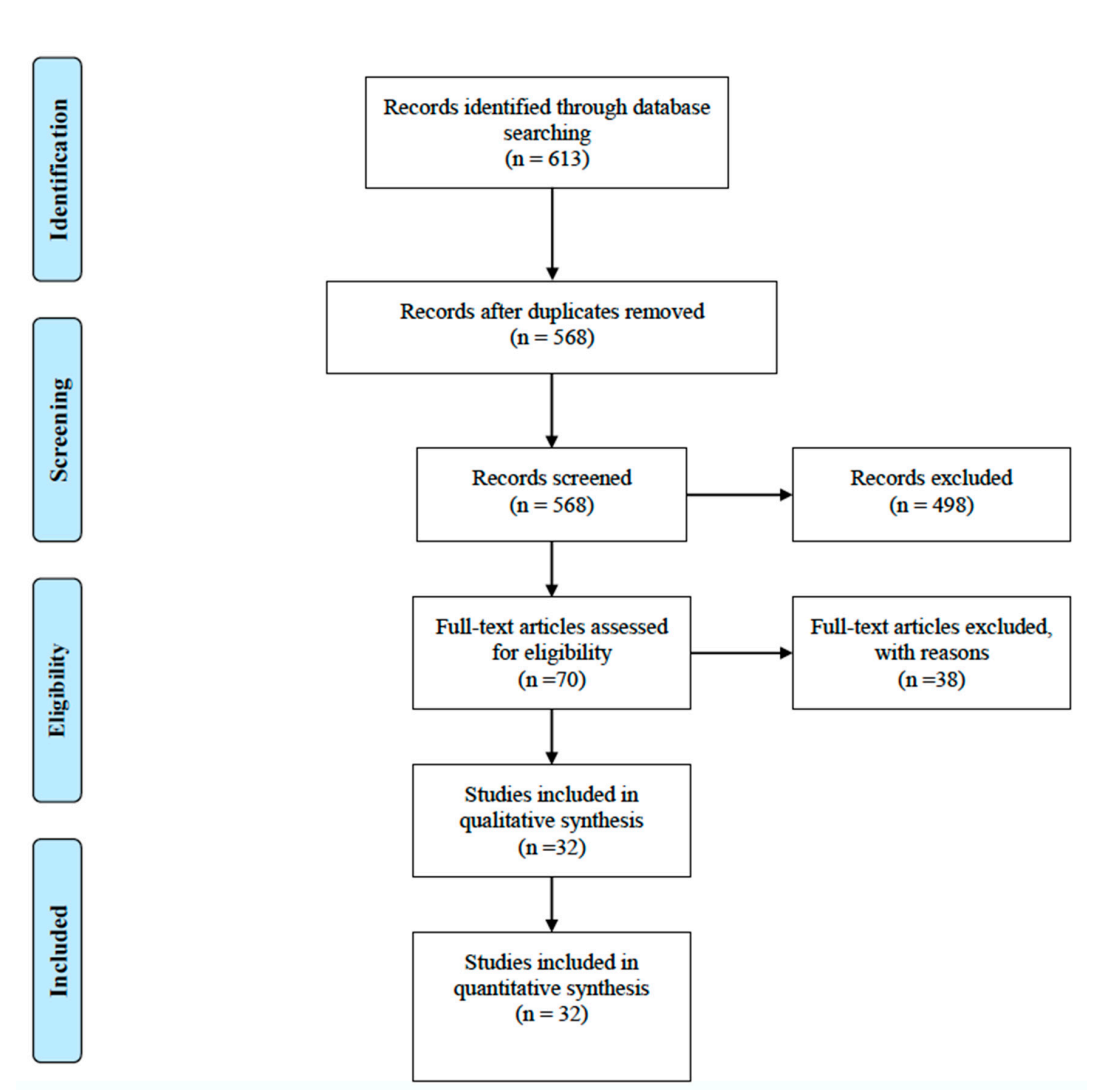
PRISMA Flow chart of the literature search.

The initial search was conducted in February 2019, with an updated search completed in August 2020. We utilized PRISMA guidelines to narrow the total amount of articles included for analysis [36]. Figure 1 depicts the decision tree for all included articles. The following databases were searched: PubMed, Scopus, SPORTDiscus, Cumulative Index to Nursing and Allied Health Literature (CINAHL), Academic Search Premier, and Cochrane Library: Central Registry of Clinical Trials. The complete list of searches conducted in the mentioned databases are listed in Appendix A. General inclusion criteria were human subjects participating in physical activity or military duty, EHS as the main injury event, studies available in the English language, and elevated body temperature due to physical exertion. Specific inclusion criteria included case series or case reports of EHS in the military and sport. Sport settings were inclusive of all skill levels, from youth to professional athletes. Military case studies included all United States military branches and militaries from countries outside the United States. Lastly, any treatment intervention for EHS that was documented in the case study and the patient outcome(s) reported by the authors were included for analysis.

### 2.2. Quality Assessment

Much of the available literature on EHS survival is documented through case reports and case series. Since it is unethical to induce EHS in human subjects, our dataset is reliant on these case studies. Our team decided to utilize a clinical case series quality assessment tool that would better suit this review. We utilized the Joanna Briggs Institute (JBI) Critical Appraisal Tools for Case Series and Case Reports [37,38,39,40], listed in Appendix B. The JBI created a set of quality assurance checklists for case series and reports to be utilized for rigor in systematic reviews [37,38]. These critical appraisal tools assess if the case report and/or series included diagnostic criteria for the patient, treatment intervention(s), and any reported adverse events [38,39,40]. The JBI Critical Appraisal Tools were the most appropriate for this review in order to select case reports and series that were thoroughly documented in order to identify risk of medical complications associated with EHS treatment interventions. Two reviewers independently scored all cases. Each reviewer was blinded in order to reduce internal bias. A third reviewer compiled all the scores and served as a tie-breaker, if necessary. Case reports were scored out of 8 points, while case series could achieve 10 points. For the purpose of this review, we required the case reports to score 6/8 and case series to score 8/10. This yielded a 75–80% quality assessment score, which the reviewers found satisfactory for analysis [39,40].

### 2.3. Descriptive Analysis

The data extracted from the available case series and reports were used to create descriptive tables for each case of EHS that met the specific inclusion criteria for this review. The variables of interest included setting (military or sport), body temperature (Tb), cooling modality, and patient outcome(s). Patient outcomes were subdivided into three categories: survived (S), survived with medical complications (SMC), and fatalities. Cases that were classified as “survived” were defined as patient(s) requiring less than 24 h of hospitalization and discharged home without secondary injuries. “Survived with medical complications”, SMC, included cases requiring hospitalization greater than 24 h and secondary injuries. Fatalities were those who succumbed to multi-system organ failure from EHS.

To examine the association between cooling methods and EHS patient outcomes, 2×2 contingency tables were constructed for cooling rate, by survival outcome(s) for the overall data set, for sport, and for military settings. All statistics were calculated with SPSS Version 26 (IBM; Armonk, NY, USA). Fisher’s Exact Test was utilized for statistical significance, *p* ≤ 0.05 without Bonferroni correction. Due to zeros in the contingency tables, 0.5 was added to offset the computational errors of zeros occurring in the calculations of risk ratios [41]. Relative risk ratios (RR) and 95% confidence intervals (CI), were then analyzed to identify the effect size and association between cooling rate and patient outcome(s). “Adequate cooling” methods in this review were defined from previous literature on exertional hyperthermia as cooling modalities with cooling rates > 0.15 °C/min [7,11]. Cooling rates slower 0.15 °C/min were defined as “insufficient” [7,11,42,43,44]. These cooling rates have been identified from previous systematic reviews and meta-analyses on exertional hyperthermia due to exercise and physical activity [7,11,42,43,44]. Since the population of interest in this review are patients competing in sporting events or participating in military duty, the cooling rates identified by McDermott et al. [11] and Zhang et al. [42] were the most appropriate to justify our definitions.

## 3. Results

**Figure 2:**
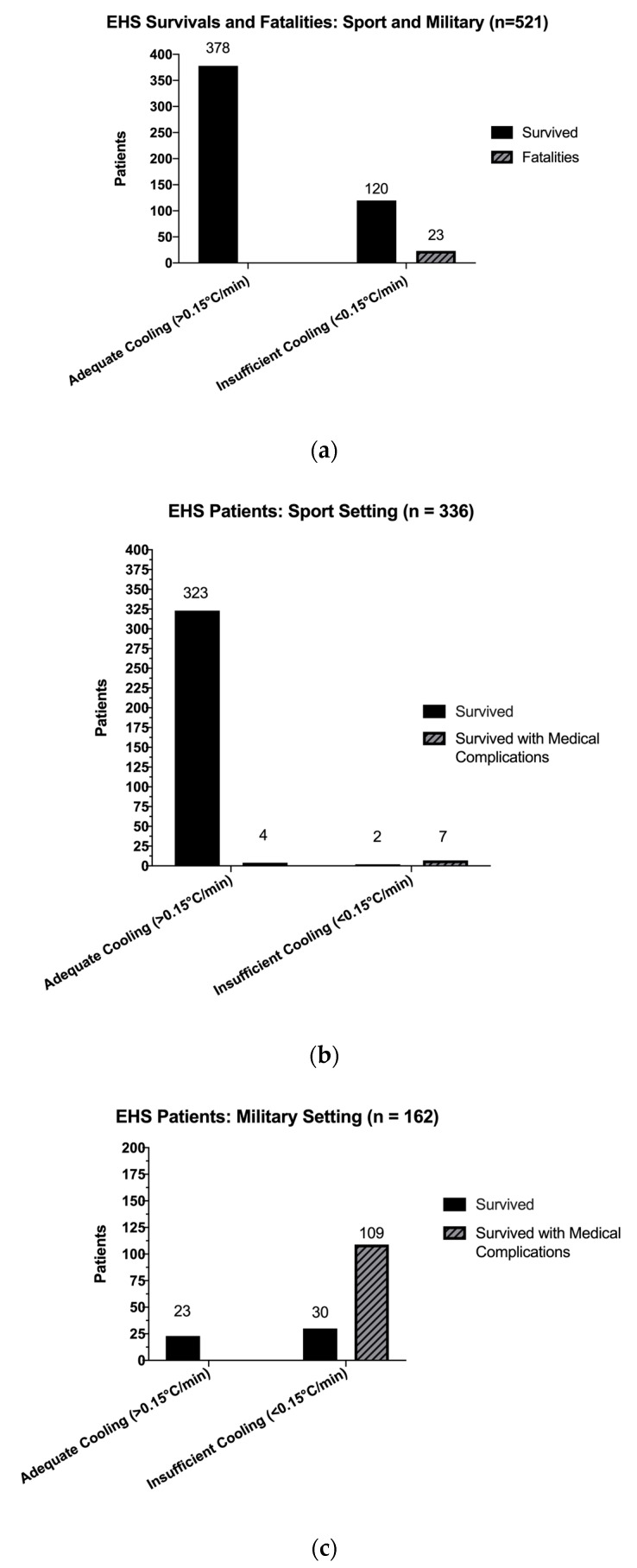
(**a**) Exertional Heat Stroke Patients in Sport and Military Settings; (**b**) Exertional Heat Stroke Patient Survivals in the Sport Setting; (**c**) Exertional Heat Stroke Patient Survivals from Military Setting.

**Figure 3:**
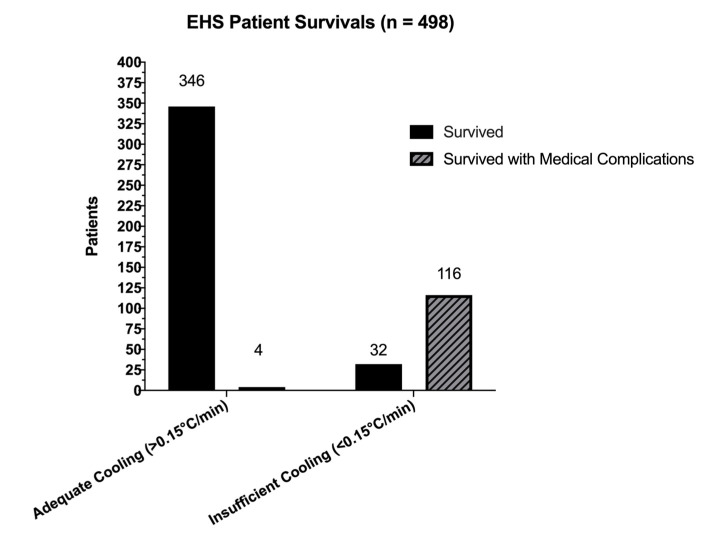
EHS Survived and Survived with Medical Complications from Sport and the Military.

Table 1 and Table 2 and Figure 2a–c include all descriptive data for the thirty-two case studies that met our inclusion criteria. Out of 521 total patients with EHS in this review, 341 (*n* = 341/521, 65.4%) patients with EHS were associated with athletic activity (Figure 2b). The military subset (Figure 2c) included 180 (*n* = 180/521, 34.55%) total patients. There were 23 fatalities in both settings combined (*n* = 23/521, 4.41%). When fatalities were removed, there were 498 survivors amongst athletes and warfighters (Figure 3), 346 survived without medical complications (*n* = 378/498, 75.9%). 

Case reports that had adequate cooling rates accounted for 378 patients (*n* = 378/521, 72.55%) in our dataset. Those who received insufficient cooling accounted for 143 patients (*n* = 143/521, 27.44%). Zero patients died (*n*= 0/521, 0.00%) who received treatment with an adequate cooling rate (Table 3). To synthesize cooling rate and the association on patient outcomes, we ran Fisher’s Exact Test without Bonferroni correction, *p* < 0.05 and calculated risk ratios for all 521 patient outcomes, then repeated tests for sport and military settings. For the entire dataset, patients who received an insufficient cooling modality had 4.57 times the risk of developing medical complications significantly associated with surviving EHS (*p* = 0.01; *p* < 0.05) than those who received a modality with an adequate cooling rate (RR = 4.57 (95%CI: 3.42, 6.28)). EHS patients in the Sport setting had 4.46 times increased risk of developing medical complications (RR= 4.46 (95% CI:1.80, 15.62)). Lastly, warfighters had 4.63 times the risk of complications if treated with an insufficient cooling rate (RR = 4.63 (95% CI: 4.57, 6.16)). Treatment with a modality with an adequate cooling rate was significantly associated with survival in both sport and military EHS patient groups (*p* = 0.01; *p* < 0.05).

## 4. Discussion

This is the first systematic review to examine EHS patient outcomes in sport and military settings. The purpose of this systematic review is to analyze survival from EHS and the influence of cooling modality rate on patient outcome(s) from sport and the military. We chose these settings because access to cooling and advanced medical care can be compromised in extreme environments if healthcare professionals are not prepared to treat EHS.

### 4.1. Patient Outcomes

Prehospital management of EHS is paramount for survival without medical complications or injury [2,5,7,8,9,10,11,12,13]. Rapid recognition, cooling, and rapid access to advanced medical care is vital for survival of EHS without medical complications [2,5,7,8,9,10,11,12,13]. In this review, “survived without medical complications” (MC) was defined as patients who survived EHS with no additional injury and were hospitalized for less than 24 h. Any patient who survived EHS, suffered secondary injuries, and required hospitalization greater than 24 h was classified as “survived with MC”. Historically, rhabdomyolysis, hepatic failure, renal failure, disseminated intervascular coagulation (DIC), encephalopathy, respiratory failure, and severe cognitive disability have been reported in patients who did not receive aggressive whole-body cooling [45,46,47,48,49,50,51,52,53,54,55,56,57,58,59,60]. The sequalae sustained from extended exercise-induced hyperthermia can be avoided when treatment with an adequate cooling rate is utilized. Quick recognition of the condition, valid and accurate body temperature assessment, and adequate cooling rates were used in 378 (*n* = 378/521, 72.55%) EHS patients that survived without MC [9,10,29,31,47,61,62,63].

### 4.2. Cooling Rate and Influence on Patient Outcomes

Our findings illustrate that EHS patients have 4.57 times the risk surviving EHS with medical complications if they are treated with a modality with an insufficient cooling rate (<0.15 °C/min) during the treatment intervention. Rapid and aggressive cooling can decrease internal body temperature below 40 °C in 30 min and prevent permanent tissue damage [7,11,44]. Specific cooling modalities that had cooling rates faster than 0.15 °C/min for survivors without MC were CWI, a combination treatment of cold water dousing with ice bag massage plus fanning [9,47,61,62,63]. In contrast, patients survived EHS with MC or died from EHS when treatments included cooling rates slower than 0.15 °C/min. The modalities with insufficient cooling rates identified in this review include the following: ice packs on arteries, ice sheet(s), fanning with spray, cold intravenous fluids, hospital cooling blankets, tepid water sponging, and fanning alone, all associated with cooling rates slower than 0.15 °C/min [11]. Dousing patients continuously with cold water and or patients submerged in cold water was a key finding of our review. Water has great potential for heat transfer because of its features such as high specific heat capacity (4.18 J·g^−1^·K^−1^), density (0.9922 g·cm^−3^) and thermal conductivity (630.5 mW·m^−1^·K^−1^) [61]. The continuous dousing or stirring of water removes the microenvironment of heat, dissipating from the patients’ skin while submerged in CWI [7,61,62]. Unfortunately, CWI is misunderstood and thought to impede heat dissipation because, in the first 5–10 min of treatment, vasoconstriction of cutaneous vessels may occur [7,11,63,64]. However, brief vasoconstriction from being immersed in cold water is negligible due to the large temperature gradient present between the patient and the water in an EHS case [7,11,61,62]. No case study present in this review demonstrated attenuated cooling rates from brief vasoconstriction of peripheral vessels. CWI as a treatment modality for EHS has been documented in the literature to decrease body temperature between 0.15–0.35 °C/min [7,11,62,63]. One hundred percent (*n* = 348/348, 100%) of patients in this data set who received CWI in our review were cooled below a body temperature of 40 °C and survived without MC [9,16,46,47,55]. Using a rapid cooling modality with an adequate cooling rate (>0.15 °C/min) is critical to prevent athletes and warfighters from severe tissue damage, secondary injury, or death induced by EHS [7,8,9,11]. 

### 4.3. Implications for Practice

Thoroughly designed treatment protocols, policies and procedures in all settings for EHS should include a modality with a cooling rate faster than 0.15 °C/min. Maximizing survival without MC includes treating the patient with CWI prior to transport to the ED. As previously mentioned, CWI has documented cooling rates of 0.15–0.35 °C/min [11]. Prehospital cooling and reducing the time a patient remains hyperthermic is critical to surviving EHS [2,3,4,5]. Collaboration amongst emergency department, military, and sports medicine healthcare providers should be made a priority in order to successfully treat patients with EHS, prior to the incident occurring. “Cool First, Transport Second” is the recommended strategy when planning for an EHS event [2,3,5,7,64]. Modalities that are transportable faster than 0.15 °C/min (e.g., ice sheets, ice packs, cold saline IV fluids, fans) have very poor cooling rates (0.028–0.078 °C/min) and will not have the same systemic effect as CWI [11]. From our dataset, using a cooling modality with a cooling rate faster than 0.15 °C/min gives the patient the best chance to survive without MC. Despite all of the literature on CWI as the optimal treatment modality for EHS, some settings do not incorporate it into field practice [45,46,47,48,49,50,51,52,53,54,55,56,57,58,59,60]. One of the counterarguments against CWI includes the logistics of the treatment intervention. An alternative to CWI in the field involves utilizing tarp-assisted cooling methods [5,65,66,67]. Two randomized control studies utilizing the tarp-assisted cooling oscillation (TACO) method demonstrated cooling rates of 0.14–0.17 °C/min [5,65,66]. While these cooling rates are not as fast as CWI, practitioners should plan to obtain a tarp, ample water, and ice as a feasible alternative to CWI in a remote location. Modalities that maximize body surface area covered with ice water can provide quick and systemic cooling of an EHS patient.

### 4.4. Limitations

The data included in this review relied on case series or reports of EHS in the literature. We could not include laborers in this review due to the lack of published data on EHS survivals in the laborer setting. Currently, it is unknown how many warfighters suffer complications from EHS; only total numbers of diagnosed EHS are reported. The diagnosis is documented as “heat stroke/sunstroke” utilizing International Classification of Diseases (ICD) 9-10 coding (ICD-9: 992.0, ICD-10: T67.0), which does not differentiate the event from exertional heat stroke [68]. Case series or reports are traditionally classified as Level IV or Grade C by the Oxford Centre for Evidence-Based Medicine and Strength of Recommendation Taxonomy (SORT) Criteria, respectively [69,70]. Since it is unethical to induce EHS and experiment with different cooling modalities in human subjects, case reports and/or series currently provide the best available evidence for treatment options in an EHS scenario. Patients that did not have well-defined treatment interventions or patient outcomes (*n* = 13/521) resulted in patients classified as survived with MC. Additionally, if the case report/series did not define treatment procedures or diagnostic criteria, those cases were listed as “not reported” in our descriptive analysis. Publication bias is also one of the limitations of this review. There are many cases of EHS in sport and the military; however, we could only analyze those case reports and series based on published, peer-reviewed literature. Therefore, it proved to be difficult to know the exact procedure for treating EHS in those specific case reports. 

## 5. Conclusions

The purpose of this systematic review was to synthesize the influence cooling modality has on the prevalence of survival with and without medical complications in EHS patients from sport and military populations. When cooling rates faster than 0.15 °C/min were present in EHS patient treatments, zero patients succumbed to EHS. Cooling modalities that have been defined as insufficient, based on previously published cooling rate literature, accounted for one hundred seventeen patients (117/521, 22.46%) surviving EHS with medical complications. Only four patients (4/521, 0.77%) that received adequate cooling had secondary injuries from EHS. Furthermore, cooling rates were significantly associated with surviving EHS without medical complications. Thorough planning, innovative solutions for challenging environments, and implementing a modality with an adequate cooling rate, specifically CWI, can reduce the number of patients requiring hospitalization for longer than 24 h and life-threatening, or fatal, outcomes from EHS. The sequalae sustained from extended exercise-induced hyperthermia can be avoided when treatment with an adequate cooling rate is utilized.

## Figures and Tables

**Table 1:** Descriptive Analysis of Exertional Heat Stroke (EHS) Cases in Military.

Author, Year	Military Location	Tb Method	Tb	Cooling Modality	Patient Outcomes (*n*)			Total Patients (*n*)
					Fatality	S	SMC	
Rav-Acha et al., 2004	Israel Defense Force	NR	NR	None (*n*= 4); Water dousing + IV (*n* = 2)	6			6
Venuto et al., 2011	U.S. Air Force	Rectal	42.22 °C	NR	1			1
Parnell and Restall, 1986	British Armed Forces	Rectal and Esophageal	Rectal = 40 °C, Esophageal = 42.8 °C	Tepid water sponging + Fan+ Cooling blanket	1			1
Shibolet et al., 1967	Israel Defense Force	NR	Mean Tb 41.0 °C	NR	8	28		36
Sithinamsuwan et al., 2009	Thailand Military	NR	Median Tb 41.6 °C	Ice packs + Water dousing + Fan + Cooling blanket + Cold spray	2		26	28
McDermott et al., 2009	U.S. Marines	Rectal	41.44 ± 0.71 °C	Cold water dousing + Ice bag massage		9		9
Beller and Boyd, 1975	U.S. Army	Rectal	42.0 ± 0.2 °C	CWI		13		13
Rohe, 2012	U.S. Marines	Rectal	41 °C	Ice packs on arteries + Cold water dousing + Fan		1		1
Barthel, 1990	U.S. Army	NR	41.1 °C	Tepid water sponging + Fan + Ice Massage		1		1
Stearns et al., 2016	U.S. Marines	Rectal	41.1 °C	CWI		1		1
	U.S. Marines	Rectal	41.2 °C	Ice Packs + Cooling Blanket			1	1
Johnston and Donham, 2012	U.S. Army, Special Forces	NR	40.5 °C *	Ice packs + Fan			1	1
Deshwal et al., 2017	India Special Forces	Rectal	41.41 ± 0.88 °C	Ice packs+ Fan + Cold spray + Cold Saline IV + O_2_			78	78
Bursey et al. 2019	U.S. Army	Rectal	43.1 °C	Ice Sheeting			1	1
Lew et al., 2002	U.S. Army	Oral	41.4 °C	None			1	1
Stewart and Whitford, 2015	U.S. Army	Rectal + Oral	42.27 °C; 42.1 °C	None			1	1

Abbreviations: Tb, Body Temperature; NR, Not Reported; S, Survived with no medical complications; SMC, Survived with medical complications; F, Fatality; CWI, Cold Water Immersion; IV, Intravenous administration; O_2_, Supplemental Oxygen Administration. * Treatment time not reported prior to transport to emergency department.

**Table 2:** Descriptive Analysis of EHS Cases in Sports.

Author, Year	Specific Activity	Tb Method	Tb	Cooling Modality	Patient Outcomes (*n*)			Total Patients (*n*)
					F	S	SMC	
Whitworth and Wolfman, 1983	Marathon (42.2 km)	NR	40 °C	Ice packs	1			1
Grundstein et al. 2016	American Football	NR	42.67 °C **	None	1			1
Asserraji et al., 2015	Marathon (42.2 km)	NR	37.5 °C *	None	1			1
Rae et al., 2008	Cycling	Rectal	41.2 °C	NR	1			1
	Cycling	Rectal	41.8 °C	NR	1			1
	Cycling	Rectal	42 °C	Cold water dousing + Fan		1		1
Armstrong et al., 1995	Road Race (11.26 km)	Rectal	40.7 ± 0.6 °C	Air exposure + Ice Towels		5		5
	Road Race (11.26 km)	Rectal	41.7 ± 0.2 °C	CWI		14		14
Whitcar et al., 2007	Recreational Run (9.6 km)	NR	39.3 °C	Cold IV fluids + fan		1		1
DeMartini et al., 2015	Road Race (11.26 km)	Rectal	41.44 ± 0.63 °C	CWI		274		274
Adams et al., 2016	Road Race (11.26 km)	Rectal	42.05 °C	CWI + Ice Towels		1		1
Sloan et al., 2015	Marathon (42.2 km)	Rectal, Oral, Tympanic	40.80 ± 2.4 °C	CWI + IV Fluids + Ice packs + Ice Towels †		29	3	32
Raj et al., 2013	Hiking	NR	41.67 °C	IV fluids			1	1
Kurowski et al., 2016	Wrestling	Oral	40.5 °C	CWI ‡,*			1	1
Takahashi et al., 2005	Rugby	Rectal	42 °C	IV fluids			1	1
Trujlio et al., 2009	Hiking	Oral	39 °C	None			1	1
Giercksy et al., 1999	Road Race (5 km)	NR	42.1 °C	None			1	1
Lopez et al., 2018	American Football	Oral	39.6 °C	None			1	1
Carvalho et al., 2016	Marathon (42.2 km)	Tympanic	39.6 °C	NR			1	1
Yue et al., 2009	Recreational Run (4.8 km)	Rectal	42.2 °C	Surgical: Cold Hemofiltration‡			1	1

Abbreviations: Tb, Body Temperature; NR, Not Reported; S, Survived with no medical complications; SMC, Survived with medical complications; F, Fatality; CWI, Cold Water Immersion; IV, Intravenous administration; O_2_, Supplemental Oxygen Administration. * Tb recorded 360 min post-collapse; ** Tb recorded 60 min post-collapse; † Treatment outcomes were not clear for patients who received CWI; ‡ Treatment initiated 210 min after admission to emergency department.

**Table 3:** Survival and Fatality from EHS for Sport and the Military by Cooling Rate.

	Survived, *n*	Fatalities, *n*	Total *n*, (%)
Adequate Cooling Rate (>0.15 °C/min)	378	0	378 (72.55%)
Insufficient Cooling Rate (<0.15 °C/min)	120	23	143 (27.44%)
Total *n*, (%)	498	23	521 (100.00%)
**a**. Survived and Survived with Medical Complications, all settings.			
	**Survived, *n***	**Survived with Medical Complications, *n***	**Total *n*, (%)**
Adequate Cooling Rate (>0.15 °C/min)	346	4	350 (70.28%)
Insufficient Cooling Rate (<0.15 °C/min)	32	116	148 (29.72%)
Total *n*, (%)	378	120	498 (100.00%)
**b**. Patient Survival from EHS in Sport.			
	**Survived, *n***	**Survived with Medical Complications, *n***	**Total *n*, (%)**
Adequate Cooling Rate (>0.15 °C/min)	323	4	327 (97.32%)
Insufficient Cooling Rate (<0.15 °C/min)	2	7	9 (2.68%)
Total *n*, (%)	325	11	336 (100.00%)
**c**. Patient Survival from EHS in the Military			
	**Survived, *n***	**Survived with Medical Complications, *n***	**Total *n*, (%)**
Adequate Cooling Rate (>0.15 °C/min)	23	0	23 (14.20%)
Insufficient Cooling Rate (<0.15 °C/min)	30	109	139 (85.80%)
Total *n*, (%)	53	109	162 (100%)

## Appendix A. Search Terms Used for the Review

**Search Engine**	**Search Number**	**Search Term**
PubMed	1	(mortal * OR death * OR died OR fatal OR fatally OR fatality OR survive OR survival OR survivor * OR saves OR saved) AND (“exertional heat stroke” OR “exercise”(mesh)OR exercise(tiab) OR “exercises” OR exercising OR postexercise OR running(mesh)OR Runner * OR running(ti) OR biker OR biking OR bicyclist * OR bicycling(mesh)OR bicycling OR bicycle * OR cyclist * OR cycling(ti) OR triathlon * OR triathlete * OR marathon * OR ultramarathon * OR “trail race” OR “trail running” OR “road race” OR “road racing” OR athlete * OR treadmill * OR ergometer * OR “endurance training” OR (“physical” AND “conditioning”) OR “speed training” OR “circuit training” OR “training duration” OR “training frequency” OR “training intensity” OR “aerobic endurance” OR “aerobic training” OR “interval training” OR “combination training” OR “combined training” OR “HIIT” OR “Sports”(mesh)OR sport(tiab) OR sports(tiab) OR military(tiab) OR “military medicine” OR “armed forces” OR army(tiab) OR navy OR marines OR “air force” OR “coast guard” OR “special forces” OR walking(mesh)OR walking(ti) OR swimming OR soccer OR football OR futbol OR “cricket” OR rugby OR Skier * OR Skiing OR Basketball OR Tennis OR Judo OR Karate OR Boxing OR Lacrosse OR “field hockey” OR Golfing OR Golf OR Hockey OR Frisbee OR quidditch OR Horseback OR Wrestling OR “Water Polo” OR Snowboarding OR “Scuba Diving” OR “Rock Climbing” OR “Martial Arts” OR Kayaking OR Hiking OR “Cross Country” OR “Adventure Race” OR “Adventure Racing” OR cyclocross OR Baseball OR gymnastics OR gymnast OR rugby OR crossfit OR “cross-fit” OR skiing OR pre-season OR preseason OR recreational OR riding OR tournament OR workout * OR “return to activity” OR “return to duty”) AND (“exertional heat stroke” OR “Heat Stroke”(mesh)OR “heat exhaustion”(mesh)OR “heat illness” OR “heat illnesses” OR “heat related illness” OR “heat related illnesses” OR “heat stroke” OR “heat strokes” OR Heatstroke * OR “EHS” OR “EHI” OR “sun stroke” OR sunstroke * OR “heat exhaustion” OR “exercise collapse” OR (collapse * AND heat) OR (collapse * AND temperature) OR “heat injury” OR “heat injuries” OR “Hyperthermia” OR Hyperthermi *) NOT ((animals(mesh)NOT humans(mesh)) OR Comment(sb)OR review(pt)OR letter(pt)OR editorial(pt)OR veterinary * OR bovine OR animal * (ti) OR pig OR pigs OR porcine OR rat OR rats OR monkey * OR mouse OR mice OR canine OR feline OR acclimate * (ti) OR acclimitizat * (ti) OR “air conditioning”(ti) OR “nursing home”(ti) OR “nursing homes”(ti) OR “non-exertional”(ti) OR “computer simulation”(mesh)OR “passive heat stress” OR “classic heat stroke” OR “classic heatstroke” OR cancer OR cancers OR neoplasm * OR MDMA OR ecstasy OR ketamine OR “Lysergic Acid” OR “music festival” OR “Music festivals” OR hernia * OR “heat shock” OR “Legal Case”[pt])
Scopus	1	TITLE-ABS-KEY(mortal * OR death * OR died OR fatal OR fatally OR fatality OR survive OR survival OR survivor * OR saves OR saved) AND (TITLE(running OR cycling) OR TITLE-ABS-KEY({exertional heat stroke} OR exercise OR exercises OR exercising OR postexercise OR post-exercis * OR Runner * OR biker OR biking OR bicyclist * OR bicycling OR bicycle * OR cyclist * OR triathlon * OR triathlete * OR marathon * OR ultramarathon * OR {trail race} OR {trail running} OR {road race} OR {road racing} OR athlet * OR treadmill * OR ergometer * OR {endurance training} OR {speed training} OR {circuit training} OR {training duration} OR {training frequency} OR {training intensity} OR {aerobic endurance} OR {aerobic training} OR {interval training} OR {combination training} OR {combined training} OR HIIT OR sport OR sports OR military OR {armed forces} OR army OR navy OR marines OR {air force} OR {coast guard} OR {special forces} OR walking OR swimming OR soccer OR football OR futbol OR cricket OR rugby OR Skier * OR Skiing OR Basketball OR Tennis OR Judo OR Karate OR Boxing OR Lacrosse OR {field hockey} OR Golfing OR Golf OR Hockey OR Frisbee OR quidditch OR Horseback OR Wrestling OR {Water Polo} OR Snowboarding OR {Scuba Diving} OR {Rock Climbing} OR {Martial Arts} OR Kayaking OR Hiking OR {Cross Country} OR {Adventure Race} OR {Adventure Racing} OR cyclocross OR Baseball OR gymnastics OR gymnast OR rugby OR crossfit OR cross-fit OR skiing OR pre-season OR preseason OR recreational OR riding OR tournament OR workout * OR {return to activity} OR {return to duty}) OR (physical w/5 conditioning)) AND TITLE-ABS-KEY({Heat Stroke} OR {heat exhaustion} OR {heat illness} OR {heat illnesses} OR {heat related illness} OR {heat related illnesses} OR {heat stroke} OR {heat strokes} OR Heatstroke * OR EHS OR EHI OR {sun stroke} OR sunstroke * OR {heat injury} OR {heat injuries} OR Hyperthermia OR Hyperthermi * OR (collapse * w/5 heat) OR (collapse * w/5 temperature) OR (exercise w/5 collapse *)) AND NOT (INDEX(MEDLINE) OR TITLE(animal * OR Comment * OR review OR letter OR editorial OR acclimat * OR acclimitizat * OR {air conditioning} OR {nursing home} OR {nursing homes} OR {non-exertional}) OR TITLE-ABS-KEY(veterinar * OR bovine OR pig OR pigs OR porcine OR rat OR rats OR monkey * OR mouse OR mice OR canine OR feline OR {computer simulation} OR {passive heat stress} OR {classic heat stroke} OR {classic heatstroke} OR cancer OR cancers OR neoplasm * OR MDMA OR ecstasy OR ketamine OR {Lysergic Acid} OR {music festival} OR {Music festivals} OR hernia * OR {heat shock})) AND (LIMIT-TO (DOCTYPE, “ar”) OR LIMIT-TO (DOCTYPE, “cp”)) AND (LIMIT-TO (SRCTYPE, “j”) OR LIMIT-TO (SRCTYPE, “p”))
SportDiscus	1	Line 1: mortal * OR death * OR died OR fatal OR fatally OR fatality OR survive OR survival OR survivor * OR saves OR saved AND Line 2: “Heat Stroke” OR “heat exhaustion” OR “heat illness” OR “heat illnesses” OR “heat related illness” OR “heat related illnesses” OR “heat stroke” OR “heat strokes” OR Heatstroke * OR EHS OR EHI OR “sun stroke” OR sunstroke * OR “heat injury” OR “heat injuries” OR Hyperthermia OR Hyperthermi * OR (collapse * AND heat) OR (collapse * AND temperature) OR (exercise AND collapse *)
	2	Line 1 (use pulldown to select title): running OR cycling OR Line2: “exertional heat stroke” OR exercise OR exercises OR exercising OR postexercise OR post-exercis * OR Runner * OR biker OR biking OR bicyclist * OR bicycling OR bicycle * OR cyclist * OR triathlon * OR triathlete * OR marathon * OR ultramarathon * OR “trail race” OR “trail running” OR “road race” OR “road racing” OR athlet * OR treadmill * OR ergometer * OR “endurance training” OR “speed training” OR “circuit training” OR “training duration” OR “training frequency” OR “training intensity” OR “aerobic endurance” OR “aerobic training” OR “interval training” OR “combination training” OR “combined training” OR HIIT OR sport OR sports OR military OR “armed forces” OR army OR navy OR marines OR “air force” OR “coast guard” OR “special forces” OR walking OR swimming OR soccer OR football OR futbol OR cricket OR rugby OR Skier * OR Skiing OR Basketball OR Tennis OR Judo OR Karate OR Boxing OR Lacrosse OR “field hockey” OR Golfing OR Golf OR Hockey OR Frisbee OR quidditch OR Horseback OR Wrestling OR “Water Polo” OR Snowboarding OR “Scuba Diving” OR “Rock Climbing” OR “Martial Arts” OR Kayaking OR Hiking OR “Cross Country” OR “Adventure Race” OR “Adventure Racing” OR cyclocross OR Baseball OR gymnastics OR gymnast OR rugby OR crossfit OR cross-fit OR skiing OR pre-season OR preseason OR recreational OR riding OR tournament OR workout * OR “return to activity” OR “return to duty” OR (physical AND conditioning)
	3	Line 1 (use pulldown to select title): animal * OR Comment * OR review OR letter OR editorial OR acclimat * OR acclimitizat * OR “air conditioning” OR “nursing home” OR “nursing homes” OR “non-exertional” OR Line 2: veterinar * OR bovine OR pig OR pigs OR porcine OR rat OR rats OR monkey * OR mouse OR mice OR canine OR feline OR “computer simulation” OR “passive heat stress” OR “classic heat stroke” OR “classic heatstroke” OR cancer OR cancers OR neoplasm * OR MDMA OR ecstasy OR ketamine OR “Lysergic Acid” OR “music festival” OR “Music festivals” OR hernia * OR “heat shock”
CINAHL	1	Line 1: mortal * OR death * OR died OR fatal OR fatally OR fatality OR survive OR survival OR survivor * OR saves OR saved AND Line 2: “Heat Stroke” OR “heat exhaustion” OR “heat illness” OR “heat illnesses” OR “heat related illness” OR “heat related illnesses” OR “heat stroke” OR “heat strokes” OR Heatstroke * OR EHS OR EHI OR “sun stroke” OR sunstroke * OR “heat injury” OR “heat injuries” OR Hyperthermia OR Hyperthermia * OR (collapse * AND heat) OR (collapse * AND temperature) OR (exercise AND collapse *)
	2	running OR cycling OR Line2: “exertional heat stroke” OR exercise OR exercises OR exercising OR postexercise OR post-exercis * OR Runner * OR biker OR biking OR bicyclist * OR bicycling OR bicycle * OR cyclist * OR triathlon * OR triathlete * OR marathon * OR ultramarathon * OR “trail race” OR “trail running” OR “road race” OR “road racing” OR athlet * OR treadmill * OR ergometer * OR “endurance training” OR “speed training” OR “circuit training” OR “training duration” OR “training frequency” OR “training intensity” OR “aerobic endurance” OR “aerobic training” OR “interval training” OR “combination training” OR “combined training” OR HIIT OR sport OR sports OR military OR “armed forces” OR army OR navy OR marines OR “air force” OR “coast guard” OR “special forces” OR walking OR swimming OR soccer OR football OR futbol OR cricket OR rugby OR Skier * OR Skiing OR Basketball OR Tennis OR Judo OR Karate OR Boxing OR Lacrosse OR “field hockey” OR Golfing OR Golf OR Hockey OR Frisbee OR quidditch OR Horseback OR Wrestling OR “Water Polo” OR Snowboarding OR “Scuba Diving” OR “Rock Climbing” OR “Martial Arts” OR Kayaking OR Hiking OR “Cross Country” OR “Adventure Race” OR “Adventure Racing” OR cyclocross OR Baseball OR gymnastics OR gymnast OR rugby OR crossfit OR cross-fit OR skiing OR pre-season OR preseason OR recreational OR riding OR tournament OR workout * OR “return to activity” OR “return to duty” OR (physical AND conditioning)
	3	Line 1 (use pulldown to select title): animal * OR Comment * OR review OR letter OR editorial OR acclimat * OR acclimitizat * OR “air conditioning” OR “nursing home” OR “nursing homes” OR “non-exertional” OR Line 2: veterinar * OR bovine OR pig OR pigs OR porcine OR rat OR rats OR monkey * OR mouse OR mice OR canine OR feline OR “computer simulation” OR “passive heat stress” OR “classic heat stroke” OR “classic heatstroke” OR cancer OR cancers OR neoplasm * OR MDMA OR ecstasy OR ketamine OR “Lysergic Acid” OR “music festival” OR “Music festivals” OR hernia * OR “heat shock”
Academic Search Premier	1	Line 1: mortal * OR death * OR died OR fatal OR fatally OR fatality OR survive OR survival OR survivor * OR saves OR saved AND Line 2: “Heat Stroke” OR “heat exhaustion” OR “heat illness” OR “heat illnesses” OR “heat related illness” OR “heat related illnesses” OR “heat stroke” OR “heat strokes” OR Heatstroke * OR EHS OR EHI OR “sun stroke” OR sunstroke * OR “heat injury” OR “heat injuries” OR Hyperthermia OR Hyperthermi * OR (collapse * AND heat) OR (collapse * AND temperature) OR (exercise AND collapse *)
	2	running OR cycling OR Line2: “exertional heat stroke” OR exercise OR exercises OR exercising OR postexercise OR post-exercis * OR Runner * OR biker OR biking OR bicyclist * OR bicycling OR bicycle * OR cyclist * OR triathlon * OR triathlete * OR marathon * OR ultramarathon * OR “trail race” OR “trail running” OR “road race” OR “road racing” OR athlet * OR treadmill * OR ergometer * OR “endurance training” OR “speed training” OR “circuit training” OR “training duration” OR “training frequency” OR “training intensity” OR “aerobic endurance” OR “aerobic training” OR “interval training” OR “combination training” OR “combined training” OR HIIT OR sport OR sports OR military OR “armed forces” OR army OR navy OR marines OR “air force” OR “coast guard” OR “special forces” OR walking OR swimming OR soccer OR football OR futbol OR cricket OR rugby OR Skier * OR Skiing OR Basketball OR Tennis OR Judo OR Karate OR Boxing OR Lacrosse OR “field hockey” OR Golfing OR Golf OR Hockey OR Frisbee OR quidditch OR Horseback OR Wrestling OR “Water Polo” OR Snowboarding OR “Scuba Diving” OR “Rock Climbing” OR “Martial Arts” OR Kayaking OR Hiking OR “Cross Country” OR “Adventure Race” OR “Adventure Racing” OR cyclocross OR Baseball OR gymnastics OR gymnast OR rugby OR crossfit OR cross-fit OR skiing OR pre-season OR preseason OR recreational OR riding OR tournament OR workout * OR “return to activity” OR “return to duty” OR (physical AND conditioning)
	3	Line 1 (use pulldown to select title): animal * OR Comment * OR review OR letter OR editorial OR acclimat * OR acclimitizat * OR “air conditioning” OR “nursing home” OR “nursing homes” OR “non-exertional” OR Line 2: veterinar * OR bovine OR pig OR pigs OR porcine OR rat OR rats OR monkey * OR mouse OR mice OR canine OR feline OR “computer simulation” OR “passive heat stress” OR “classic heat stroke” OR “classic heatstroke” OR cancer OR cancers OR neoplasm * OR MDMA OR ecstasy OR ketamine OR “Lysergic Acid” OR “music festival” OR “Music festivals” OR hernia * OR “heat shock”
Cochrane Library: CENTRAL Registry of Clinical Trials	1	Line 1 (use pulldown to select title abstract keyword): mortal * OR death * OR died OR fatal OR fatally OR fatality OR survive OR survival OR survivor * OR saves OR saved AND Line 2 (use pulldown to select title abstract keyword): “Heat Stroke” OR “heat exhaustion” OR “heat illness” OR “heat illnesses” OR “heat related illness” OR “heat related illnesses” OR “heat stroke” OR “heat strokes” OR Heatstroke * OR EHS OR EHI OR “sun stroke” OR sunstroke * OR “heat injury” OR “heat injuries” OR Hyperthermia OR Hyperthermia * AND Line 3 (use pulldown to select title abstract keyword): running OR cycling OR “exertional heat stroke” OR exercise OR exercises OR exercising OR postexercise OR post-exercis * OR Runner * OR biker OR biking OR bicyclist * OR bicycling OR bicycle * OR cyclist * OR triathlon * OR triathlete * OR marathon * OR ultramarathon * OR “trail race” OR “trail running” OR “road race” OR “road racing” OR athlet * OR treadmill * OR ergometer * OR “endurance training” OR “speed training” OR “circuit training” OR “training duration” OR “training frequency” OR “training intensity” OR “aerobic endurance” OR “aerobic training” OR “interval training” OR “combination training” OR “combined training” OR HIIT OR sport NOT Line 4 (use pulldown to select record title): animal * OR Comment * OR review OR letter OR editorial OR acclimat * OR acclimitizat * OR “air conditioning” OR “nursing home” OR “nursing homes” OR “non-exertional” OR veterinar * OR bovine OR pig OR pigs OR porcine OR rat OR rats OR monkey * OR mouse OR mice OR canine OR feline OR “computer simulation” OR “passive heat stress” OR “classic heat stroke” OR “classic heatstroke” OR cancer OR cancers OR neoplasm * OR MDMA OR ecstasy OR ketamine OR “Lysergic Acid” OR “music festival” OR “Music festivals” OR hernia * OR “heat shock”
	2	Line 1 (use pulldown to select title abstract keyword): mortal * OR death * OR died OR fatal OR fatally OR fatality OR survive OR survival OR survivor * OR saves OR saved AND Line 2 (use pulldown to select title abstract keyword): “Heat Stroke” OR “heat exhaustion” OR “heat illness” OR “heat illnesses” OR “heat related illness” OR “heat related illnesses” OR “heat stroke” OR “heat strokes” OR Heatstroke * OR EHS OR EHI OR “sun stroke” OR sunstroke * OR “heat injury” OR “heat injuries” OR Hyperthermia OR Hyperthermi * AND Line 3 (use pulldown to select title abstract keyword): sports OR military OR “armed forces” OR army OR navy OR marines OR “air force” OR “coast guard” OR “special forces” OR walking OR swimming OR soccer OR football OR futbol OR cricket OR rugby OR Skier * OR Skiing OR Basketball OR Tennis OR Judo OR Karate OR Boxing OR Lacrosse OR “field hockey” OR Golfing OR Golf OR Hockey OR Frisbee OR quidditch OR Horseback OR Wrestling OR “Water Polo” OR Snowboarding OR “Scuba Diving” OR “Rock Climbing” OR “Martial Arts” OR Kayaking OR Hiking OR “Cross Country” OR “Adventure Race” OR “Adventure Racing” OR cyclocross OR Baseball OR gymnastics OR gymnast OR rugby OR crossfit OR cross-fit OR skiing OR pre-season OR preseason OR recreational OR riding OR tournament OR workout * OR “return to activity” OR “return to duty” OR “physical conditioning” NOT Line 4 (use pulldown to select record title): animal * OR Comment * OR review OR letter OR editorial OR acclimat * OR acclimitizat * OR “air conditioning” OR “nursing home” OR “nursing homes” OR “non-exertional” OR veterinar * OR bovine OR pig OR pigs OR porcine OR rat OR rats OR monkey * OR mouse OR mice OR canine OR feline OR “computer simulation” OR “passive heat stress” OR “classic heat stroke” OR “classic heatstroke” OR cancer OR cancers OR neoplasm * OR MDMA OR ecstasy OR ketamine OR “Lysergic Acid” OR “music festival” OR “Music festivals” OR hernia * OR “heat shock”

## Appendix B. Joanna Briggs Institute Critical Appraisal Checklist for Case Reports and Case Series

Reviewer_ _ _ _ _ _ _ _ _ _ _ _ _ _Date_ _ _ _ _ _ _ _ _ _ _ _

Author _ _ _ _ _ _ _ _ _ _ _ _ _ _Year_ _ _ _ _Record Number_ _ _

	Yes	No	Unclear	Not Applicable
1. Were patient’s demographic characteristics clearly described?	□	□	□	□
2. Was the patient’s history clearly described and presented as a timeline?	□	□	□	□
3. Was the current clinical condition of the patient on presentation clearly described?	□	□	□	□
4. Were diagnostic tests or assessment methods and the results clearly described?	□	□	□	□
5. Was the intervention(s) or treatment procedure(s) clearly described?	□	□	□	□
6. Was the post-intervention clinical condition clearly described?	□	□	□	□
7. Were adverse events (harms) or unanticipated events identified and described?	□	□	□	□
8. Does the case report provide takeaway lessons?	□	□	□	□

Overall appraisal: Include □ Exclude □ Seek further info □

Comments (Including reason for exclusion)

______________________________________________

______________________________________________

______________________________________________

**Explanation of Case Reports Critical Appraisal**

*How to cite:* Moola S, Munn Z, Tufanaru C, Aromataris E, Sears K, Sfetcu R, Currie M, Qureshi R, Mattis P, Lisy K, Mu P-F. Chapter 7: Systematic reviews of etiology and risk. In: Aromataris E, Munn Z (Editors). *Joanna Briggs Institute Reviewer’s Manual.* The Joanna Briggs Institute, 2017. Available from https://reviewersmanual.joannabriggs.org/

**Case Reports Critical Appraisal Tool**

Answers: Yes, No, Unclear or Not/Applicable

1. Were patient’s demographic characteristics clearly described?

Does the case report clearly describe patient’s age, sex, race, medical history, diagnosis, prognosis, previous treatments, past and current diagnostic test results, and medications? The setting and context may also be described.

2. Was the patient’s history clearly described and presented as a timeline?

A good case report will clearly describe the history of the patient, their medical, family and psychosocial history including relevant genetic information, as well as relevant past interventions and their outcomes. (CARE Checklist 2013)

3. Was the current clinical condition of the patient on presentation clearly described?

The current clinical condition of the patient should be described in detail including the uniqueness of the condition/disease, symptoms, frequency and severity. The case report should also be able to present whether differential diagnoses was considered.

4. Were diagnostic tests or methods and the results clearly described?

A reader of the case report should be provided with sufficient information to understand how the patient was assessed. It is important that all appropriate tests are ordered to confirm a diagnosis and therefore the case report should provide a clear description of various diagnostic tests used (whether a gold standard or alternative diagnostic tests). Photographs or illustrations of diagnostic procedures, radiographs, or treatment procedures are usually presented when appropriate to convey a clear message to readers.

**JBI Critical Appraisal Checklist for Case Series**

Reviewer_ _ _ _ _ _ _ _ _ _ _ _ _ _Date_ _ _ _ _ _ _ _ _ _ _ _

Author _ _ _ _ _ _ _ _ _ _ _ _ _ _Year_ _ _ _ _Record Number_ _ _

	Yes	No	Unclear	Not Applicable
1. Were there clear criteria for inclusion in the case series?	□	□	□	□
2. Was the condition measured in a standard, reliable way for all participants included in the case series?	□	□	□	□
3. Were valid methods used for identification of the condition for all participants included in the case series?	□	□	□	□
4. Did the case series have consecutive inclusion of participants?	□	□	□	□
5. Did the case series have complete inclusion of participants?	□	□	□	□
6. Was there clear reporting of the demographics of the participants in the study?	□	□	□	□
7. Was there clear reporting of clinical information of the participants?	□	□	□	□
8. Were the outcomes or follow up results of cases clearly reported?	□	□	□	□
9. Was there clear reporting of the presenting site(s)/clinic(s) demographic information?	□	□	□	□
10. Was statistical analysis appropriate?	□	□	□	□

Overall appraisal: Include □ Exclude □ Seek further info □

Comments (Including reason for exclusion)

______________________________________________

______________________________________________

______________________________________________

**Introduction to the Case Series Critical Appraisal Tool**

*How to cite:* Moola S, Munn Z, Tufanaru C, Aromataris E, Sears K, Sfetcu R, Currie M, Qureshi R, Mattis P, Lisy K, Mu P-F. Chapter 7: Systematic reviews of etiology and risk. In: Aromataris E, Munn Z (Editors). *Joanna Briggs Institute Reviewer’s Manual.* The Joanna Briggs Institute, 2017. Available from https://reviewersmanual.joannabriggs.org/

The definition of a case series varies across the medical literature, which has resulted in inconsistent use of this term (Appendix C). The gamut of case studies is wide, with some studies claiming to be a case series realistically being nothing more than a collection of case reports, with others more akin to cohort studies or even quasi-experimental before and after studies. This has created difficulty in assigning ‘case series’ a position in the hierarchy of evidence and identifying and appropriate critical appraisal tool.

Dekkers et al. define a case series as a study in which ‘only patients with the outcome are sampled (either those who have an exposure or those who are selected without regard to exposure), which does not permit calculation of an absolute risk.’ The outcome could be a disease or a disease related outcome. This is contrasted to cohort studies where sampling is based on exposure (or characteristic), and case- control studies where there is a comparison group without the disease.

The completeness of a case series contributes to its reliability. Studies that indicate a consecutive and complete inclusion are more reliable than those that do not. For example, a case series that states ‘we included all patients (24) with osteosarcoma who presented to our clinic between March 2005 and June 2006′ is more reliable than a study that simply states ‘we report a case series of 24 people with osteosarcoma.’

For the purposes of this checklist, we agree with the principles outlined in the Dekker et al. paper, and define case series as studies where only patients with a certain disease or disease-related outcome are sampled. Some of the items below relate to risk of bias, whilst others relate to ensuring adequate reporting and statistical analysis. A response of ‘no’ to any of the questions below negatively impacts the quality of a case series.

**Tool Guidance**

Answers: Yes, No, Unclear or Not/Applicable

1. Were there clear criteria for inclusion in the case series?

The authors should provide clear inclusion (and exclusion criteria where appropriate) for the study participants. The inclusion/exclusion criteria should be specified (e.g., risk, stage of disease progression) with sufficient detail and all the necessary information critical to the study.

2. Was the condition measured in a standard, reliable way for all participants included in the case series?

The study should clearly describe the method of measurement of the condition. This should be done in a standard (i.e., same way for all patients) and reliable (i.e., repeatable and reproducible results) way.

3. Were valid methods used for identification of the condition for all participants included in the case series?

Many health problems are not easily diagnosed or defined, and some measures may not be capable of including or excluding appropriate levels or stages of the health problem. If the outcomes were assessed based on existing definitions or diagnostic criteria, then the answer to this question is likely to be yes. If the outcomes were assessed using observer reported, or self-reported scales, the risk of over- or under-reporting is increased, and objectivity is compromised. Importantly, determine if the measurement tools used were validated instruments, as this has a significant impact on outcome assessment validity.

4. Did the case series have consecutive inclusion of participants?

Studies that indicate a consecutive inclusion are more reliable than those that do not. For example, a case series that states ‘we included all patients (24) with osteosarcoma who presented to our clinic between March 2005 and June 2006′ is more reliable than a study that simply states ‘we report a case series of 24 people with osteosarcoma.’

5. Did the case series have complete inclusion of participants?

The completeness of a case series contributes to its reliability (1). Studies that indicate a complete inclusion are more reliable than those that do not. A stated above, a case series that states ‘we included all patients (24) with osteosarcoma who presented to our clinic between March 2005 and June 2006′ is more reliable than a study that simply states ‘we report a case series of 24 people with osteosarcoma.’

6. Was there clear reporting of the demographics of the participants in the study?

The case series should clearly describe relevant participant’s demographics such as the following information where relevant: participant’s age, sex, education, geographic region, ethnicity, time period, education.

7. Was there clear reporting of clinical information of the participants?

There should be clear reporting of clinical information of the participants such as the following information where relevant: disease status, comorbidities, stage of disease, previous interventions/treatment, results of diagnostic tests, etc.

8. Were the outcomes or follow-up results of cases clearly reported?

The results of any intervention or treatment should be clearly reported in the case series. A good case study should clearly describe the clinical condition post-intervention in terms of the presence or lack of symptoms. The outcomes of management/treatment when presented as images or figures can help in conveying the information to the reader/clinician. It is important that adverse events are clearly documented and described, particularly if a new or unique condition is being treated or when a new drug or treatment is used. In addition, unanticipated events, if any, that may yield new or useful information should be identified and clearly described.

9. Was there clear reporting of the presenting site(s)/clinic(s) demographic information?

Certain diseases or conditions vary in prevalence across different geographic regions and populations (e.g., women vs. men, sociodemographic variables between countries). The study sample should be described in sufficient detail so that other researchers can determine if it is comparable to the population of interest to them.

10. Was statistical analysis appropriate?

As with any consideration of statistical analysis, consideration should be given as to whether there was a more appropriate alternate statistical method that could have been used. The methods section of studies should be detailed enough for reviewers to identify which analytical techniques were used and whether these were suitable.

## Appendix C. Case Series Definitions

‘A report on a series of patients with an outcome of interest. No control group is involved.’(^24^) (p. 279)

‘A case series is a descriptive study involving a group of patients who all have the same disease or condition: the aim is to describe common and differing characteristics of a particular group of individuals’ (Oxford Handbook of medical statistics)

‘A group or series of case reports involving patients who were given similar treatment. Reports of case series usually contain detailed information about the individual patients. This includes demographic information (for example, age, gender, ethnic origin) and information on diagnosis, treatment, response to treatment, and follow-up after treatment.’ Law K, Howick J. OCEBM Table of Evidence Glossary. 2013 [cited 2014 10th January]; Available from: http://www.cebm.net/index.aspx?o=1116

‘A case series (also known as a clinical series) is a type of medical research study that tracks subjects with a known exposure, such as patients who have received a similar treatment, or examines their medical records for exposure and outcome.’ Wikipedia

‘A study which makes observations on a series of individuals, usually all receiving the same intervention, with no control group. Comments: At this stage it is unclear whether case series should be included in Cochrane systematic reviews, but we have left them in the list so that working groups can consider whether there are circumstances in which it would be appropriate to include them, and to assess risk of bias. A particular reason for including case series might be where they provide evidence relating to adverse effects of an intervention. Potential examples of risk of bias might be that if a case series does not [attempt to] recruit consecutive participants, this might introduce a risk of selection bias, while some case series could be at risk of detection bias, if the circumstances in which adverse effects are reported (or elicited) are not standardized.’ http://bmg.cochrane.org/research-projectscochrane-risk-bias-tool
